# Age-associated reduction of cell spreading induces mitochondrial DNA common deletion by oxidative stress in human skin dermal fibroblasts: implication for human skin connective tissue aging

**DOI:** 10.1186/s12929-015-0167-6

**Published:** 2015-07-28

**Authors:** Chunji Quan, Moon Kyun Cho, Daniel Perry, Taihao Quan

**Affiliations:** Department of Pathology, Affiliated Hospital of Yanbian University, Yanji, Jilin Province People’s Republic of China; Department of Dermatology, Soonchunhyang University College of Medicine, Seoul, South Korea; Department of Dermatology, University of Michigan Medical School, 1301 Catherine, Medical Science I, Room 6447, Ann Arbor, MI 48109-5609 USA

**Keywords:** Cell shape, Mitochondrial common deletion, Reactive oxygen species, Human skin connective tissue aging

## Abstract

**Background:**

Reduced cell spreading is a prominent feature of aged dermal fibroblasts in human skin *in vivo*. Mitochondrial DNA (mtDNA) common deletion has been reported to play a role in the human aging process, however the relationship between age-related reduced cell spreading and mtDNA common deletion has not yet been reported.

**Results:**

To examine mtDNA common deletion in the dermis of aged human skin, the epidermis was removed from full-thickness human skin samples using cryostat. mtDNA common deletion was significantly elevated in the dermis of both naturally aged and photoaged human skin *in vivo.* To examine the relationship between age-related reduced cell spreading and mtDNA common deletion, we modulated the shape of dermal fibroblasts by disrupting the actin cytoskeleton. Reduced cell spreading was associated with a higher level of mtDNA common deletion and was also accompanied by elevated levels of endogenous reactive oxygen species (ROS). Boosting cellular antioxidant capacity by using antioxidants was found to be protective against mtDNA common deletion associated with reduced cell spreading.

**Conclusion:**

mtDNA common deletion is highly prevalent in the dermis of both naturally aged and photoaged human skin *in vivo*. mtDNA common deletion in response to reduced cell spreading is mediated, at least in part, by elevated oxidative stress in human dermal fibroblasts. These data extend current understanding of the mitochondrial theory of aging by identifying the connection between mtDNA common deletion and age-related reduction of cell spreading.

## Background

Human skin is the largest organ of the human body. Its functions include providing a protective barrier from environmental stressors including heat, solar ultraviolet (UV) irradiation, infection, injury, and water loss. Human skin, like all other organs, undergoes a natural aging process with time. Unlike other organs, human skin also continuously experiences harmful stress from environmental sources such as solar UV irradiation that leads to damage [1, 2]. Based on its causes, cutaneous aging is classified into two types: natural aging and photoaging [3, 4]. Natural aging refers to those changes observed in all individuals resulting from the passage of time, whereas photoaging refers to those changes attributable to habitual sun exposure. Both of these processes are cumulative, therefore photoaging is superimposed on intrinsic aging.

Histological and ultrastructural studies have revealed that the major alterations in aged skin are localized to the dermal connective tissue and are manifested as a thin and damaged dermis [5, 6]. These features are derived directly from deleterious alterations in collagen, the most abundant structural protein in skin. Alterations of the collagenous ECM microenvironment directly relate to the development of age-related skin pathologies by causing increased fragility, impaired vasculature support, poor wound healing, and a tissue microenvironment that promotes epithelial cancer [7–9].

In human skin, dermal fibroblasts are responsible for collagen homeostasis. Consequently, impaired dermal fibroblast function is a major contributing factor in human skin connective tissue aging [10–12]. We previously reported that a prominent characteristic of dermal fibroblasts in aged skin is reduced spreading and contact with collagen fibrils, causing cells to lose their typical elongated spindle-like morphology and become shorter with a rounded and collapsed morphology [6, 13, 14]. In young healthy skin, dermal fibroblasts attach to intact collagen fibrils and achieve normal cell spreading and shape. However, in aged dermis the collagen fibrils are fragmented, which impairs fibroblast-collagen interactions. These alterations impair fibroblast spreading and function. While cell shape is known to regulate many cellular functions [15–18], the molecular basis of their impact on dermal fibroblast function and skin connective tissue aging are not well understood.

Mitochondrial DNA (mtDNA) mutations and deletions have long been proposed to play a role in many human diseases [19–22] as well as the aging process [23–27]. mtDNA common deletion (4977 bp) is one of the best described mitochondrial deletions [22, 28]. mtDNA deletions including mtDNA common deletion have been reported to be prevalent in aged human skin [2, 23, 26, 29]. However, these existing data largely describe the levels of mtDNA deletions in epidermal keratinocytes, and the high turnover of keratinocytes would result in little time to accumulate mtDNA deletion. Although dermal fibroblasts are the major cell type responsible for the maintenance of dermal connective tissue homeostasis, little is known about the role of mtDNA common deletion in aging dermal fibroblasts. Dermal fibroblasts have a very low proliferative rate which would allow for an accumulation of mtDNA deletion. Additionally, the relationship between age-related reduced cell spreading, which is a prominent feature of aged dermal fibroblasts, and mtDNA common deletion has been virtually unexplored. Based on this information, we explored the possible connection between age-related reduced cell spreading and mtDNA common deletion in the dermis of human skin. We found that mtDNA common deletion is significantly increased in both naturally aged and photoaged human skin dermis *in vivo*, and that reduced fibroblast spreading induces the increase in mtDNA common deletion through increased endogenous reactive oxygen species (ROS). This mechanism provides new insight into the connection between age-related reduced cell spreading and the mtDNA common deletion/ROS axis, implicating a molecular basis for the pathophysiology of human skin connective tissue aging.

## Methods

### Procurement of human skin samples

Punch biopsies from sun-protected human buttock skin were obtained from clinically normal adult volunteers; 20–30 year-old males for the young group (N = 8, mean age 24 ± 3 years) and 80+ year-olds for the aged group (N = 8, mean age 82 ± 4 years). Samples of severely photoaged skin and subject-matched sun-protected skin were obtained from the extensor forearm and sun-protected underarm, respectively (N = 10, mean age 54 ± 3 years). The presence of severe photodamage was determined based on clinical criteria as previously described [30]. Skin samples were 4 mm diameter sections of full thickness skin. All procedures involving human subjects were approved by the University of Michigan Institutional Review Board, and all subjects provided written informed consent.

### Cell culture

Primary adult human dermal fibroblasts were isolated from punch biopsies of sun-protected buttock skin from healthy volunteers (mean age 31 ± 4 years) [31]. Briefly, dermal fibroblasts were isolated by digesting skin with bacterial collagenase (Worthington Biochemical Corporation, Lakewood, NJ, USA). Early passage primary adult human dermal fibroblasts (less than nine passages) were cultured in Dulbecco’s Modified Eagle’s Media (DMEM, Invitrogen Life Technology, Carlsbad, CA, USA) with 10 % Fetal Calf Sera (FBS, Invitrogen Life Technology, Carlsbad, CA, USA) at 37 °C, 5 % CO_2_. The approximate population doubling time was 2 days. For latrunculin-A (Lat-A) treatment, cells were treated with Lat-A at a concentration of 30 nM for three days. For some studies, cultured dermal fibroblasts were treated with N-acetylcysteine (NAC, Sigma, St Louis, MO, USA) at a concentration of 10 mM immediately after Lat-A treatment for three days.

### RNA quantitative real-time RT-PCR

RNA isolation and quantitative real-time RT-PCR were performed as previously described [31]. Briefly, total RNA was extracted by Trizol reagent and 100 ng total RNA was reverse transcribed using a Taqman Reverse Transcription Kit (Applied Biosystems, Foster City, CA, USA). Real-time RT-PCR was performed by SYBR green real-time PCR using a 7300 Sequence Detector (Applied Biosystems, Foster City, CA, USA). Human keratin 14, type I procollagen, and 36B4 primers were described previously [32]. Target gene mRNA were normalized to the housekeeping gene 36B4 (a ribosomal protein used as an internal control for quantification).

### Quantitative determination of mtDNA with 4977 bp deletion

**Fig. 1:**
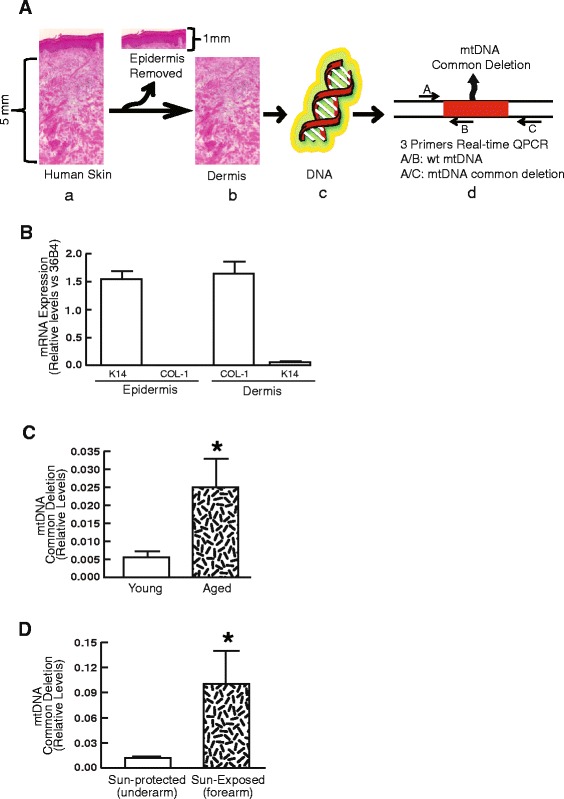
Accumulation of mtDNA common deletion in the dermis of naturally aged and photoaged human skin *in vivo.* **a** Schematic representation of the dissection of human skin dermis and detection of mtDNA common deletion in the dermis. a) Haematoxylin & Eosin staining of full-thickness human skin; b) Skin dermis was prepared by cutting off epidermis at a depth of 1 mm by cryostat; c) Purified total DNA from the dermis; d) Detection of mtDNA common deletion in the dermis. Real-time QPCR primers A, B, and C (indicated by arrows) were used to simultaneously PCR DNA fragments from both wild-type mtDNA (primers A/B) and deleted mtDNA (primers A/C). The PCR fragment from primers A/C was too large for wildtype mtDNA (>5 kb) to be amplified with the PCR conditions used for detection (see *Methods* for details). **b** Minimal contamination of keratinocyte in the dermis. Total RNA was prepared from epidermal and dermal sections, and keratin 14 (marker of epidermis) and type I procollagen (marker of dermal fibroblasts) mRNA levels were quantified by real-time RT-PCR. Keratin 14 and type I procollagen mRNA levels were normalized by the housekeeping gene (36B4, internal control). Mean ± SEM. N = 6, *p < 0.05. **c** mtDNA common deletion is increased in the dermis of naturally aged human skin *in vivo.* mtDNA common deletion was determined by real-time QPCR (see details for *Methods*). Young group (N = 8, mean age 24 ± 3 years) and aged group (N = 8, mean age 82 ± 4 years). Mean ± SEM. *p < 0.05. **d** mtDNA common deletion is increased in the dermis of photoaged human skin *in vivo.* mtDNA common deletion was determined by real-time QPCR (see *Methods* for details). Photoaged skin and subject matched sun-protected skin were obtained from the extensor forearm and sun-protected buttocks, respectively. Mean ± SEM. N = 10. *p < 0.05

Genomic DNA from human skin dermis and *in vitro* tissue culture cells was isolated using QIAamp DNA Minikit (QIAGEN, Inc., Hilden, Germany). To quantify mtDNA common deletion (4,977-bp deletion in mtDNA), we employed the quantitative “3 primer PCR” method [24, 33], which simultaneously detects both wild-type and common deletion, as illustrated in Fig. 1a. Oligonucleotide primers B and C corresponded to heavy strand positions 13720–13705 and 9028–9008, respectively; primer A corresponded to light strand positions 8273–8289. Primer B was designed in the region of mtDNA common deletion, whereas primers A and C flanked the region of mtDNA common deletion. By design, nested primers A and B only amplified wild-type mtDNA (755 bp), whereas nested primers A and C only amplified deleted mtDNA (470 bp) [33]. Primers A and C would not yield any PCR products if there was no deleted mtDNA, because the large flanking region (>5-kb) is too long to be amplified under PCR conditions of very short polymerase extension time, as described below. The PCR reaction mixture (25 μl total volume) contained 150 ng DNA template, 2 μM PCR primers, 10 μM dNTPs, and PCR master mix (Applied Biosystems, Foster City, CA, USA). The PCR conditions were pre-denaturation at 94 °C for 2 min, followed by 35 cycles at 94 °C for 25 s, 60 °C for 30s, and a final extension at 72 °C for 10 min. These PCR products were confirmed by electrophoresis on a 2 % agarose gel and sequencing (data not shown) [24, 33]. The levels of deleted and wild-type mtDNA were measured by real-time PCR using a 7300 Sequence Detector (Applied Biosystems, Foster City, CA, USA). The mtDNA common deletion levels (primers A/C) were normalized by simultaneously measuring wild-type mtDNA (primers A/B). PCR was performed and normalized against a serial dilution of a control DNA sample, and then each target PCR product in the samples was quantified based on the corresponding standard curve. All PCR experiments included a negative control with no template DNA (double-distilled water) and a positive control with PCR products from primers A and C (470 bp), which was cloned into pCDNA 3.1 (Invitrogen, Carlsbad, CA, USA).

### Phalloidin staining, intracellular ROS measurements, and mitochondrial stain

Cell morphology was assessed by incubating cultures with Phalloidin. Cells were washed with PBS and fixed in 2 % paraformaldehyde for 30 min followed by Phalloidin stain (Sigma, St. Louis, MO, USA) for one hour. Relative cell surface areas were quantified by ImageJ (NIH, MD, USA). Intracellular ROS was measured by redox-sensitive fluorescent dye (Redox Sensor Red CC-1, Grand Island, NY, USA). Briefly, fibroblasts were incubated with Redox Sensor Red CC-1 (1 μM) at 37 °C for one hour in the dark. Cells were washed twice in PBS, then fixed with 2 % paraformaldehyde for 15 min. The cells were stained with DAPI to visualize nuclei, and cells were observed and photographed using Zeiss HBO100 fluorescence microscope. Intracellular ROS was quantified by ImageJ (NIH, MD, USA). Briefly, the intensity of the red area corresponding to oxidized RedoxSensor Red CC-1-positive staining was measured from 10 random fields per slide. Next, the software settings were programmed to quantify all nuclei (DAPI, blue staining) in the same fields, and the number of cells analyzed was considered. Finally, the intensity of the red area (indication of ROS levels) was normalized by the number of cells and the results expressed as a relative ROS level. To stain mitochondria, cells were incubated with MitoTracker® fluorescent dye (200 nM for 30 min, Molecular Probes, Inc.Eugene, OR, USA) according to the manufacture’s protocol. MitoTracker® passively diffuses across the plasma membrane and accumulates in active mitochondria of live cells.

### Statistical analysis

Statistical significance between groups was determined with the Student’s *t*-test. All p values are two-tailed and considered significant when p < 0.05.

## Results

### mtDNA common deletion is accumulated in the dermis of naturally-aged and photoaged human skin *in vivo*

To assess mtDNA common deletion in the dermis, the epidermis was removed using cryostat to cut at a depth of 1 mm (Fig. 1a). To monitor separation of epidermis from dermis, the levels of keratin 14 mRNA (a marker of basal keratinocytes) and type I procollagen mRNA (a marker of dermal fibroblasts) were determined in each fraction. Keratin 14 mRNA levels in the dermal fraction were reduced 82-fold relative to the epidermis, indicating minimal contamination from keratinocytes in the dermal fraction, most likely from deeper appendages such as hair follicles and sweat glands (Fig. 1b). In contrast, type I procollagen mRNA levels were 152-fold greater in the dermis relative to epidermis, indicating collagen-rich dermis (Fig. 1b).

We next determined the levels of mtDNA common deletion in the dermis by purification of DNA from the dermis. As shown in Fig. 1c, the levels of mtDNA common deletion were significantly increased in aged human skin dermis compared to young skin dermis. The average level of mtDNA common deletion in aged dermis was 4.5-fold greater compared to young skin dermis. Similarly, the levels of mtDNA common deletion were much higher in sun-exposed photodamaged skin dermis (forearm) than in sun-protected skin dermis (underarm) (Fig. 1d). The average level of mtDNA common deletion in photodamaged skin dermis was 6.9-fold larger compared to sun-protected skin dermis. These results demonstrate the accumulation of mtDNA common deletion in the dermis of both naturally aged and photoaged human skin *in vivo*.

### Reduced cell spreading induces mtDNA common deletion associated with increased reactive oxygen species (ROS) in human skin dermal fibroblasts

**Fig. 2:**
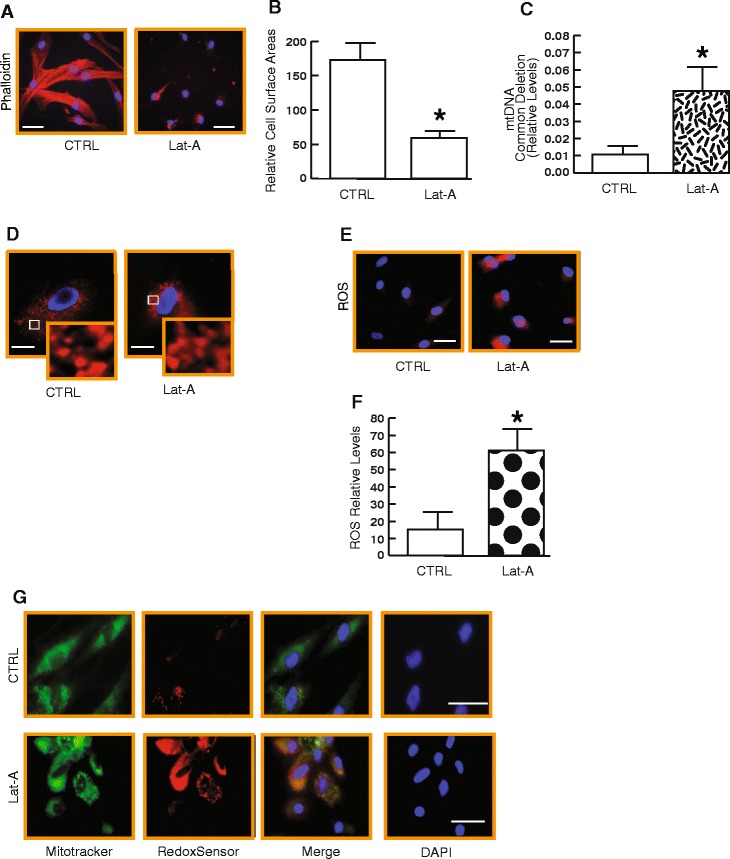
Reduced cell spreading induces mtDNA common deletion associated with increased reactive oxygen species (ROS) in human skin dermal fibroblasts. **a** Representative images of dermal fibroblasts treated with Lat-A (see *Methods* for details), which impairs actin polymerization and fibroblast spreading resulting in a rounded cell shape. Dermal fibroblasts were stained with phalloidin and were imaged by fluorescence microscopy. Red fluorescence delineates cell cytoplasm; blue fluorescence delineates nuclei. Bars = 50 μm. **b** The relative cell surface areas were quantified by ImageJ. Mean ± SEM, N = 4, *p < 0.05. **c** Reduced cell spreading induces mtDNA common deletion. mtDNA common deletion was determined by real-time QPCR (see details for *Methods*). Mean ± SEM. N = 4 *p < 0.05. **d** Cells were stained with MitoTracker fluorescent dye and were imaged by fluorescence microscopy. Red fluorescence delineates gross morphology of mitochondria; blue fluorescence delineates nuclei. Bars = 50 μm. **e** and **f** Reduced cell spreading induces ROS. Intracellular ROS levels were measured by Redox Sensor Red fluorescence and quantified by ImageJ (see *Methods* for details). Red fluorescence indicates ROS and blue fluorescence delineates nuclei. Mean ± SEM. N = 4, *p < 0.05. **g** Reduced cell spreading induces mitochondrial ROS. Cells were double stained with Mitotracker (green) and RedoxSensor (red); blue fluorescence delineates nuclei. Bars = 100 μm

We next explored the possible connection between age-related reduced cell spreading, which is a prominent feature of aged dermal fibroblasts, and mtDNA common deletion in dermal fibroblasts from human skin. We modulated the shape of dermal fibroblasts by disrupting the actin cytoskeleton with latrunculin-A (Lat-A), which rapidly blocks actin polymerization [34]. As expected, disruption of the actin cytoskeleton impaired fibroblast spreading and resulted in a rounded shape (Fig. 2a, right panel). Staining of the actin cytoskeleton with phalloidin indicated a loss of actin cytoskeletal fibers and a reduced cell area (Fig. 2a, right panel). Quantification indicated that the surface areas of fibroblasts were reduced by 63 % in Lat-A treated cells compared to control cells (Fig. 2b). Interestingly, reduced cell spreading was associated with a significant elevation of mtDNA common deletion (Fig. 2c). The levels of mtDNA common deletion were increased 3.2-fold by reduced cell spreading (Fig. 2c). These data suggest that reduced cell spreading leads to elevated mtDNA common deletion in human dermal fibroblasts**.** As mitochondrial morphology is crucial for normal mitochondrial function, we assessed mitochondrial morphology by labeling mitochondria with MitoTracker fluorescent dye. These data indicated that the gross shape of mitochondria was similar between Lat-A treated cells and control cells (Fig. 2d). It has been reported that cellular damage from reactive oxygen species (ROS) likely plays an important role in mtDNA deletions as well as in the aging process [25, 35, 36]. We therefore examined the relative oxidant levels in fibroblasts using the redox-sensitive fluorescent dye RedoxSensor Red CC-1. Normal well-spreading fibroblasts displayed a very low level of oxidant-generated fluorescence (Fig. 2e, left panel). In contrast, reduced-spreading fibroblasts displayed intense oxidant-generated fluorescence (Fig. 2e, right panel). The level of oxidant-generated fluorescence was 4.2-fold greater in fibroblasts that had reduced spreading than in well-spreading fibroblasts (Fig. 2f). RedoxSensor Red is oxidized by a broad range of oxidizing species, therefore it reflects a general cellular redox state. To identify the source of endogenous ROS, the cells were double stained with mitochondrial markers Mitotracker and RedoxSensor. Figure 2g revealed a strong co-localization of Mitotracker and RedoxSensor, indicating that the mitochondria are the primary source of ROS induced by reduced cell spreading. Taken together, these data indicate that reduced cell spreading, due to disassembly of the actin cytoskeleton, leads to increased mtDNA common deletion accompanied by elevated mitochondrial ROS levels.

### Antioxidant treatment protects against mtDNA common deletion induced by reduced cell spreading in human dermal fibroblasts

**Fig. 3:**
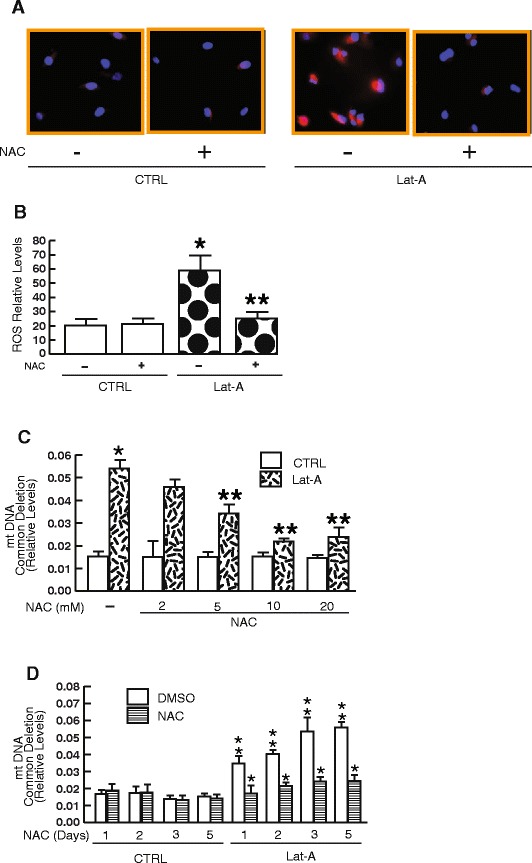
Antioxidant treatment protects against reduced-cell-spreading-associated mtDNA common deletion in human dermal fibroblasts. **a** and **b** Fibroblasts were treated with vehicle (CTRL, DMSO) or N-acetylcysteine (NAC, 10 mM) immediately after Lat-A treatment for three days. Intracellular ROS levels were measured by Redox Sensor Red fluorescence and quantified by ImageJ (see *Methods* for details). Red fluorescence indicates ROS and blue fluorescence delineates nuclei. Mean ± SEM. N = 4, *p < 0.05 vs CTRL. ** p < 0.05 vs Lat-A without NAC. **c** Anti-oxidant treatment protects against elevated mtDNA common deletion by oxidative exposure in a dose-dependent manner. Fibroblasts were treated with vehicle (CTRL, DMSO) or N-acetylcysteine (NAC, 2–20 mM) immediately after Lat-A treatment for three days. mtDNA common deletion was determined by real-time QPCR (see *Methods* for details). Mean ± SEM. *p < 0.05 vs CTRL. ** p < 0.05 vs Lat-A without NAC. **d** Time-course of anti-oxidant protection against mtDNA common deletion by oxidative exposure. Fibroblasts were treated with vehicle (CTRL, DMSO) or N-acetylcysteine (NAC, 10 mM) immediately after Lat-A treatment for indicated times. mtDNA common deletion was determined by real-time QPCR (see *Methods* for details). Mean ± SEM. *p < 0.05 vs Lat-A without NAC. ** p < 0.05 vs without Lat-A and NAC

We next investigated whether boosting cellular antioxidant capacity could protect against mtDNA common deletion associated with reduced cell spreading. We chose N-acetyl-cysteine (NAC), which is an antioxidant and metabolic precursor of glutathione [37]. Glutathione serves as a co-factor for the antioxidant enzyme glutathione peroxidase, which plays a critical role in protecting cells from oxidative damage by reducing lipid peroxides and converting hydrogen peroxide to water. Treatment of fibroblasts with NAC (10 mM) for three days immediately after Lat-A treatment markedly diminished the elevation of endogenous ROS levels (Fig. 3a). Treatment of fibroblasts with NAC effectively reduced ROS levels by 87 % in Lat-A treated cells (Fig. 3b). We next determined whether the addition of NAC could prevent the negative influence of oxidative stress on the levels of mtDNA common deletion associated with reduced cell spreading. NAC treatment partially but significantly prevented the elevated levels of mtDNA common deletion in a dose-dependent manner (Fig. 3c). Figure 3d further demonstrated that reduced cell spreading increased mtDNA common deletion in a time-dependent manner, and that the increase was significantly prevented by NAC treatment. These results indicate that the deleterious effects of endogenous oxidative exposure are responsible, at least in part, for reduced-cell-spreading-associated mtDNA common deletion.

## Discussion

Human skin is largely composed of collagen-rich connective tissue, which provides structural and functional support. The collagen-rich connective tissue is produced, organized, and maintained by dermal fibroblasts. During aging, dermal collagen fibrils undergo progressive loss and fragmentation, leading to thin and structurally weakened skin. Age-related alterations of collagen fibrils impair skin structure and function, and create a tissue microenvironment that promotes age-related skin problems. Fibroblasts in young human dermis appear flattened and spread, and are in intimate contact with numerous intact collagen fibrils. In contrast, fibroblasts in aged human dermis have a collapsed appearance with little cytoplasm, and lack direct association with fragmented collagen fibrils [6, 13, 14]. Reduced fibroblast spreading has significant consequences in human skin connective tissue aging. Firstly, reduced fibroblast spreading significantly up-regulates matrix metalloproteinase-1 (MMP-1) expression through the activation of AP-1 transcription factor, a major driving force for MMP-1 expression [38]. Age-related elevation of MMP-1 causes fragmentation of collagen fibrils, which is considered an important process in human skin connective tissue aging [12, 38, 39]. Secondly, reduced fibroblast spreading significantly down-regulates collagen production through impairment of TGF-β signaling [10, 12, 40], which is a primary regulator of collagen and other ECM protein production [10, 41]. Reduced production of collagen and other ECM proteins contributes significantly to thinning of the skin, a prominent feature of aged human skin.

We recently demonstrated that increasing dermal fibroblast spreading through improved structural support of the dermal ECM microenvironment can activate fibroblasts to a more "youthful" state in aged human skin *in vivo* [40, 42]. Injection of dermal filler (cross-linked hyaluronic acid) into the skin of individuals over 70 years of age enhances cell spreading, leading to stimulated fibroblast collagen production and proliferation, expanded vasculature, and increased epidermal thickness. Therefore, fibroblasts in aged human skin retain their capacity for functional activation, which can be restored by enhancing cell spreading. These data also indicate that proliferation and function of other cell types, including endothelial cells and keratinocytes, can be enhanced in aged skin by enhancing cell spreading. These findings support the concept that cell spreading, along with tissue microenvironment, is critical for dermal fibroblast function.

In this report we show another aspect of the deleterious effect of age-related reduced dermal fibroblast spreading on skin connective tissue aging, through induction of mtDNA common deletion. Although the accumulation of mtDNA common deletion in aged human skin has been reported for more than two decades [23, 26, 29], our data provide two pieces of new information that give us a better understanding of the age-related accumulation of mtDNA common deletion in skin. First, we demonstrate that mtDNA common deletion is induced by age-related reduction of dermal fibroblast spreading. Our findings extend current understanding of the mitochondrial theory of aging by identifying reduction of cell spreading as a factor for age-related accumulation of mtDNA common deletion. Secondly, we demonstrate that mitochondria are the primary source of elevated ROS in response to age-related reduction of cell spreading. There are multiple sources of intracellular ROS in mammals, including mitochondria, NADPH oxidases (NOX), xanthine oxidase, monoamine oxidase, and nitric oxide synthase. Mitochondrial ROS are widely associated with several age-related chronic diseases and the health of many vital organ systems [43–45]. Although beyond the scope of this study, it is of interest to investigate whether mitochondrial ROS are associated with reduced cell spreading in age-related chronic diseases. It is also of interest to test whether mitochondrial-targeted antioxidants, such as MitoQ and SkQ1, effectively protect against mitochondrial damage induced by reduced cell spreading.

Accumulating evidence suggests that increased mtDNA mutations/deletions can lead to increased ROS production, while at the same time oxidative stress can also induce mtDNA mutations/deletions [36, 46–50]. Our data demonstrates that mtDNA common deletion in response to reduced cell spreading is mediated, at least in part, by elevated ROS, suggesting the possibility of a positive feedback loop between ROS and mtDNA common deletion in response to reduced cell spreading. The mechanism by which reduced cell spreading leads to increased endogenous ROS levels remains to be determined. Cell shape impacts a multitude of cellular processes including signal transduction, gene expression, and metabolism [15–18]. Recent evidence suggests that cytoskeletal tension plays a key role in translation of mechanical information into cell function [51–53]. In general, intracellular redox homeostasis is maintained by enzymatic antioxidant defenses such as superoxide dismutase (SOD), glutathione peroxidase (GPx), and catalase (CAT). SODs are responsible for the dismutation of superoxide radicals, generated by NAD(P)H oxidases, to hydrogen peroxide. CAT converts hydrogen peroxide into water and oxygen. Impairment or imbalance of the enzymatic antioxidant defenses could contribute to elevated ROS generation in response to reduced cell spreading. We are currently investigating the possibility of whether an imbalance of antioxidant enzyme levels may contribute to elevated ROS levels in dermal fibroblasts in response to reduced spreading. Currently, knowledge regarding the relationship among reduction of cell spreading, ROS, and mtDNA common deletion is in a nascent state. Further studies are needed to understand the mechanisms that couple reduced cell spreading to oxidative stress and mtDNA common deletion in human dermal fibroblasts, their role in skin connective tissue aging, and possible therapeutic implications. In addition, future work should focus on types of mtDNA deletions/mutations other than common deletion, which may be considerably more prevalent.

Accumulating evidence suggests that aging is associated with increased frequency of mitochondrial DNA mutations/deletions and increased production of ROS [36, 46–50]. However, such observations have been made primarily in tissues with high rates of oxidative metabolism such as brain and muscle. The extent to which mtDNA mutations/deletions contribute to age-related changes in skin dermis remains to be clarified. Although an increased frequency of mtDNA mutations has been reported in naturally and photoaged aged human skin [2, 23, 24, 26], the frequency of mtDNA common deletion in the dermal compartment in naturally and photoaged skin has not been well documented. Our results demonstrate the accumulation of mtDNA common deletion in the dermis of both naturally and photoaged human skin *in vivo*. These data are consistent with our previous report that oxidant levels are indeed increased in dermal fibroblasts in aged skin *in vivo* [38]. The magnitude of mtDNA common deletion seems to be 10-fold higher in photoaged skin (Fig. 1d) compared to naturally aged skin (Fig. 1c), which is consistent with the general concept that more severe dermal connective tissue damage occurs in photoaged skin than in naturally aged skin.

Antioxidant treatments to either retard the aging process or to treat age-related disorders are subjects of heightened interest. NAC is safe for human use, and we previously demonstrated that NAC penetrates human skin and effectively mitigates ROS-driven responses to acute UV irradiation in human skin *in vivo* [54]. Our data suggest that antioxidant NAC may be able to retard skin connective tissue aging through reduction of mtDNA common deletion. Slowing the accumulation of mtDNA common deletion would likely result in less functional decline in dermal fibroblasts over time. It is tempting to speculate that the combination of an antioxidant and an agent that promotes features of cell spreading would promote normal dermal fibroblast function and revitalize skin.

## Conclusion

**Fig. 4:**
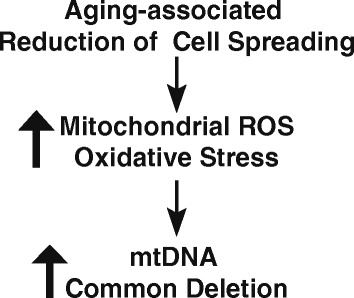
Proposed model for age-related reduction of dermal fibroblast spreading induces mtDNA common deletion through ROS/oxidative stress (see *Discussion* for details)

In conclusion, based on our findings in aged human skin dermis *in vivo* and *in vitro*, we postulate that age-related reduction of dermal fibroblast spreading brings about numerous alterations including increased ROS/oxidative stress, which promotes mtDNA common deletion (Fig. 4). Increased mtDNA common deletion could further induce ROS/oxidative stress through a positive feedback mechanism, forming a critical mechanism of human skin aging. This mechanism extends current understanding of the oxidative theory of aging by recognizing that age-related reduction of dermal fibroblast spreading induces mtDNA common deletion through ROS/oxidative stress.

## Abbreviations

mtDNA: mitochondrial DNA
Lat-A: Latrunculin-A
ROS: Reactive oxygen species
NAC: N-acetyl-cysteine
UV: Ultraviolet
COL-1: type I collagen
K14: Keratin 14
MMP-1: Matrix metalloproteinase 1
ECM: Extracellular matrix
PCR: Polymerase chain reaction
AP-1: Activator protein-1
